# Comparison of Rural vs Urban Direct-to-Physician Commercial Promotion of Medications for Treating Opioid Use Disorder

**DOI:** 10.1001/jamanetworkopen.2019.16520

**Published:** 2019-12-02

**Authors:** Thuy Nguyen, Barbara Andraka-Christou, Kosali Simon, W. David Bradford

**Affiliations:** 1 O’Neill School of Public and Environmental Affairs, Indiana University, Bloomington; 2 Department of Health Management and Informatics, University of Central Florida, Orlando; 3 National Bureau of Economic Research, Cambridge, Massachusetts; 4 Department of Public Administration and Policy, University of Georgia, Athens, Georgia

## Abstract

**Importance:**

In the United States, access to medications prescribed for opioid use disorder (OUD) is lower in rural counties than in urban counties. Considering the positive associations between direct-to-physician promotion of opiates and OUD medications and their prescribing rates, a study examining the association between pharmaceutical promotion of these medications and county-level rurality has merit.

**Objective:**

To assess whether rural counties received less pharmaceutical promotion of OUD medications compared with urban counties.

**Design, Setting, and Participants:**

This cross-sectional county-level study used all reported direct-to-physician pharmaceutical payments from manufacturers of medications prescribed for OUD from January 1, 2014, through December 31, 2017, as well as demographic and economic data at the county level from 3140 US counties. Logistic regression was used with year and state-level fixed effects to compare rural county and urban county odds of receiving any promotion of OUD medications. A negative binomial model was used with year and state-level fixed effects to compare the mean pharmaceutical payments per physician and per population in rural vs urban counties.

**Main Outcomes and Measures:**

A binary indicator for whether physicians in a county received any promotion related to OUD medications in a year. The second outcome was the value of promotion (eg, meals), with dollar amount of payments for each county by year. Counties were separated into metropolitan, micropolitan, and rural categories using the National Center for Health Statistics Urban-Rural Classification Scheme.

**Results:**

Of 3140 US counties with 18 318 physicians to whom promotion of OUD medications was directed, 1166 (37.1%) were metropolitan (16 740 physicians [91.4%]), 641 (20.4%) were micropolitan (1049 physicians [5.7%]), and 1333 (42.5%) were rural (529 physicians [2.9%]). Compared with physicians in metropolitan counties, physicians in rural counties had reduced odds of receiving any promotion (adjusted odds ratio, 0.57; 95% CI, 0.44-0.74) and received lower payments (adjusted incidence rate ratio, 0.24; 95% CI, 0.17-0.34).

**Conclusions and Relevance:**

The study findings suggest that promotion for OUD medications is less likely to occur in rural counties and that this difference in promotion of OUD medications may be associated with differential commercial costs and benefits of promotion in rural settings.

## Key Points

**Question:**

Is there an association between county-level rurality and direct-to-physician commercial promotion of medications prescribed for opioid use disorder?

**Findings:**

In this cross-sectional study of 3140 US counties from 2014 to 2017, among 18 318 physicians to whom promotion of opioid use disorder medications was directed, rural counties were less likely to receive any promotion, and receive lower payments compared with those in urban counties.

**Meaning:**

The findings suggest that the commercial promotion of medications prescribed for opioid use disorder is less likely to occur in rural counties and that the difference may be associated with differential commercial costs and benefits of promotion in these areas.

## Teaser

This cross-sectional study compares direct-to-physician commercial promotion of medications for opioid use disorder in rural vs urban county locations.

## Introduction

Fewer than 20% of people with opioid use disorder (OUD) receive empirically validated treatment.^1^ Medications prescribed for OUD, including methadone, buprenorphine, and naltrexone, have been found to be associated with decreased mortality and morbidity from OUD.^2,3,4^ These medications are recommended as part of treatment for OUD by professional health care organizations and US government agencies, including the Substance Abuse and Mental Health Services Administration (SAMHSA)^5^ and the American Society of Addiction Medicine.^6^ Methadone can only be dispensed in federally regulated opioid treatment programs, which are primarily located in urban areas.^7^ The shortage of opioid treatment programs is particularly acute in rural areas.^8^ The OUD medications buprenorphine and naltrexone, unlike methadone, may be prescribed outside highly regulated opioid treatment programs (eg, in solo practitioner offices). However, prescribers of buprenorphine require a waiver from SAMHSA and must adhere to patient limits; these requirements do not apply to prescribers of naltrexone.

Despite the effectiveness of OUD medications, 71% of rural counties lack a publicly listed prescriber of these medications.^9^ In addition, most health care clinicians with a SAMHSA waiver prescribe buprenorphine hydrochloride to fewer patients than allowed under the federal patient limit.^10^ Because of the ongoing opioid overdose crisis, it appears that a combination of actions from a wide range of stakeholders is needed to increase prescribing of OUD medications. It is unlikely that actions by any single stakeholder will be sufficient to address treatment gaps,^11^ given the many factors associated with insufficient OUD medication prescribing, including stigma toward patients, lack of knowledge and training among health care clinicians, and limited resources (eg, staff time).^5^ Instead, a combination of actions by many stakeholders may be necessary to expand use of OUD medications, potentially including actions by for-profit corporations.

Marketing promotion initiated by pharmaceutical manufacturers to health professionals typically occurs when drug company representatives make sales calls or visits to medical practitioners and supply them with drug information, meals, gifts, or free samples.^12^ Pharmaceutical promotion, accounting for the highest proportion of marketing spending by drug companies,^13^ is a possible route toward expanding access of OUD medications, but this route has been underexplored in the scientific literature. Previous work suggests that these marketing efforts are associated with reducing the time to adopt new drugs^14^ but also raises concerns regarding the association of these efforts with prescribing practices among clinicians.^15^ One type of pharmaceutical promotion is direct payments from pharmaceutical companies to physicians (direct-to-physician payments), such as for food and beverages or for speaking engagements.^16^ All drug companies are required to publicly disclose such direct-to-physician payments to a government database, according to the 2010 Physician Payments Sunshine Act.^17,18^

A recent study^19^ found that promotion of OUD medications, which primarily comes from manufacturers of branded medications, was significantly associated with increased medication prescribing. Though causal relationships were not examined in that study,^19^ it may be possible to conclude that the promotion increased the willingness of physicians to prescribe OUD medications. At a minimum, pharmaceutical promotion may serve as a mechanism for addressing well-known information gaps about the treatment of OUD among physicians.^20^ Given the lower availability of medications prescribed for OUD in rural areas, we examined geographic disparities in pharmaceutical promotion of OUD medications. Despite rural areas having experienced a disproportionate burden of hospitalizations associated with prescription opioid overdoses,^21^ profit incentives for pharmaceutical companies to market OUD medications may be lower in rural areas compared with urban areas because of smaller population sizes and greater travel distances. We hypothesized that the frequency of promotion and payments of medications prescribed for OUD has been lower in rural areas.

## Methods

### Study Design

This cross-sectional study used all reported direct-to-physician pharmaceutical payments of OUD medications as well as demographic and economic data. In this study, we used longitudinal data on 3140 US counties from January 1, 2014, to December 31, 2017. The raw data recording pharmaceutical payments are mandated to be publicly available according to the 2010 Sunshine Act^18^ and include identifiable information about physicians. This secondary data analysis was determined to be exempt from review and informed consent by the institutional review board of the Indiana University Human Subjects Office because there was no interaction with participants. Only locations of physicians were used to identify which county received promotion. This study followed the Strengthening the Reporting of Observational Studies in Epidemiology (STROBE) reporting guideline.

### Data Sources

Information on direct-to-physician pharmaceutical payments (monetary and monetary equivalent gifts) for OUD medications was obtained from the Sunshine Act’s Open Payments data^22^ from January 1, 2014, through December 31, 2017; these data are collected and published online by the Centers for Medicare & Medicaid Services. We extracted all payment records that mentioned at least 1 drug name and aggregated these payments to the 5-digit zip code level. Zip codes were mapped to their corresponding Federal Information Processing Standards (FIPS) county codes using the ZIP-FIPS crosswalk file in the R package noncensus (R Foundation for Statistical Computing).^23,24^ Counties without any reported payment records were assumed to receive no payments from manufacturers of medications prescribed for OUD.

County-level rurality or urbanicity was categorized as metropolitan, micropolitan, and rural using the National Center for Health Statistics Urban-Rural Classification Scheme.^9^ In particular, we used the most rural category, identified as nonmetropolitan noncore counties, to define rural counties in this study. The metropolitan category included counties in the metropolitan statistical area of a million or more population. The remaining counties were categorized as micropolitan. We used data on the number of active physicians (ie, any physician; this number was used as a weight in the analysis) and primary care physicians (a physician subset used as a control variable in this analysis) from the Health Resources and Services Administration Area Health Resources Files.^25^ Data on active physicians were not available in 2014; thus, we proxied with 2015 data; for 2017, data from 2016 were used.

Demographic and economic data including race/ethnicity, household income, population, and proportion of older individuals were collected from the Robert Wood Johnson Foundation County Health Rankings file.^26^ The proportion of adults aged 19 to 64 years who were insured was collected from the US Census Bureau’s Small Area Health Insurance Estimates program.^27^ We estimated the number of opioid-related deaths in a county per 100 000 residents from the National Vital Statistics System of the Centers for Disease Control and Prevention Multiple Cause of Death files.^28^ The number of buprenorphine-waivered clinicians was obtained from the Drug Enforcement Agency Active Controlled Substances Act Registrants listing^29^ of the National Technical Information Service;^29^ we were only able to obtain 2017 data. We extracted data on the number of substance abuse treatment facilities from the National Survey of Substance Abuse Treatment Services files.^30^ All county information was lagged by 1 year in our analysis unless otherwise noted. We used lagged time-variant control variables such as opioid-related mortality rates and insured rates to ensure that these predictors of pharmaceutical promotion were determined before the occurrence of such promotion.

### Outcome

The first outcome variable that we examined was a binary indicator for whether any physician in a county received an OUD medication–related payment in a year. The second outcome was the dollar amount of these payments in each county by year. To account for the disparity in the population size of physicians and patients in rural vs urban counties, we weighted this dollar amount by the number of physicians who received OUD medication–related payments (recipients) in our main analysis. Dollar amounts in this context were considered as count of dollars received in a county. This variable was rounded to an integer in our count models. Count regressions model the log of incident counts; therefore, the incidence rate ratios (IRRs) were reported instead of the regression coefficients for more straightforward interpretations. In the sensitivity analysis, we calculated the payment amounts in US dollars per 1000 physicians in a county and the payment amounts per 100 000 residents.

Medications prescribed for OUD in this analysis consisted of buprenorphine hydrochloride or both buprenorphine and naloxone hydrochloride (Bunavail [BioDelivery Sciences International], Suboxone [Reckitt Benckiser Pharmaceuticals Inc], Probuphine [Titan Pharmaceuticals Inc], Zubsolv [DJA Global Pharmaceuticals Inc], and generic forms of buprenorphine-naloxone [Amneal Pharmaceuticals and Roxane Laboratories]) and extended-release naltrexone (Vivitrol [Alkermes Inc]) based on previous work.^31^ Pharmaceutical payments for the recent US Food and Drug Administration approval of buprenorphine extended-release (Sublocade [Indivior Inc]) and buprenorphine and naloxone sublingual film (Cassipa [Teva Pharmaceuticals USA Inc]) as well as buprenorphine alone (Subutex [Indivior Inc]) were not found in the data for our study period.

### Control Variables

The selection of control variables in this study was based on previous literature.^9,19^ Mean income and insurance coverage (insured adults aged 19-64 years and the population share of Medicare beneficiaries) were included as variables associated with the ability to pay for OUD medications. We also controlled for overall population and the number of primary care physicians as a proxy for market size. Furthermore, sales representatives of OUD medications may have targeted communities with higher opioid overdose–related mortality. The number of buprenorphine-waivered clinicians (both physician and nonphysician) and substance abuse treatment facilities, the only authorized locations to prescribe buprenorphine, were also used as controls. For example, our data showed that 7.1% of counties without buprenorphine-waivered clinicians received promotion for OUD medications in 2017, whereas 63.2% of counties with such waivers received any promotion. Using an alternative source of buprenorphine prescribers (the SAMHSA locator file archived in 2017), we also found that 10.5% of counties without publicly listed prescribers of OUD medications received promotions, whereas 65.7% of counties with such prescribers received promotions.

### Statistical Analysis

Descriptive statistics of outcomes were calculated and compared across the 3 categories of population size. A logistic regression with year and state-level fixed effects was used to compare rural county and urban county odds ratios (ORs) of receiving promotion of OUD medications. Including state-level fixed effects could have helped control for state-level time-invariant factors, but within a logistic model, it would have dropped data from 2 states. In a logistic regression, adding state fixed effects would drop states without variance in the dependent variable across time and county: 1 county in the District of Columbia and 3 counties in Delaware. Instead, we estimated a linear probability model because the addition of state fixed effects does not drop any states in that case; we reported similar results from this exercise as those from our main logistic regression model. A negative binomial model with year and state-level fixed effects was used for comparing the mean pharmaceutical payment amounts of OUD medications per recipient in rural vs urban counties. The negative binomial model was selected instead of a Poisson model or linear regression because of the skewed distribution of payments.

These regressions controlled for demographic factors associated with pharmaceutical promotion mentioned above. Using county-level longitudinal data might lead to dependency of the outcome from 1 year to another. Of 940 counties that had promotions in 2014, 796 continued to have promotions in 2017. The within variation in likelihood of receiving promotions was 0.34 and the between variation was 0.31. We did not control for county-level fixed effects, such as in a typical county-level longitudinal analysis, which would drop any time-invariant variable (such as rurality) in the model. Although we had longitudinal data, the panel was too short to reliably estimate fixed effects or any time dependency in the error term. Thus, we corrected for within-cluster error correlation by county and provided cluster-robust 95% CIs. We performed regression analysis using Stata, version 16.0 (StataCorp). The 95% CIs were used for all statistical tests (including Wald tests for coefficients and 2-sided *t* tests for group means). The statistical significance threshold was 5%, and significance testing was 2-sided.

## Results

### Participants

Of 3140 US counties with 18 318 physicians to whom promotion of OUD medications was directed, 1166 (37.1%) were metropolitan (16 740 physicians [91.4%]), 641 (20.4%) were micropolitan (1049 physicians [5.7%]), and 1333 (42.5%) were rural (529 physicians [2.9%]). We obtained all payments of OUD medications reported in the Sunshine Act database^18^ from January 1, 2014, through December 31, 2017. The number of recipients of these payments substantially increased from 6591 physicians in 2014 to 10 657 physicians in 2017. The total payment amount during this period was $9.6 million.

### Descriptive Data

**Figure 1.**
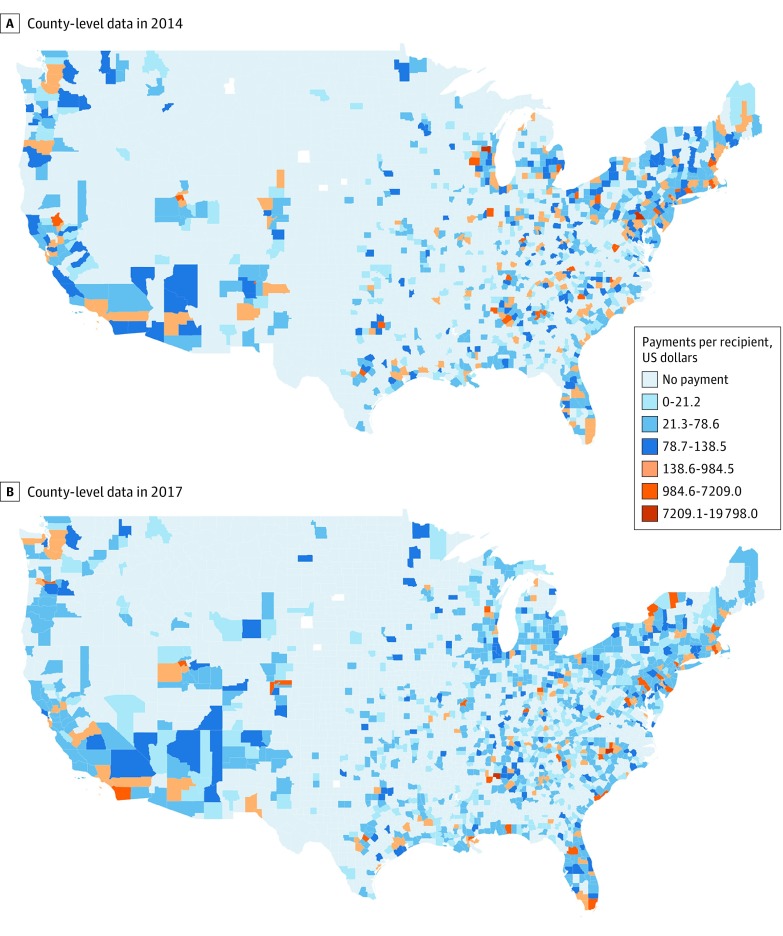
US Counties Receiving Any Promotion of Medication for Opioid Use Disorder (OUD) County-level data showing pharmaceutical promotion of OUD medication from the Sunshine Act’s Open Payments repository. The data brackets are based on percentile values of payments for OUD medication per recipient in 2014 (A) and 2017 (B): $0 to $21.20 (75th percentile), $21.30 to $78.60 (90th percentile), $78.70 to $138.50 (95th percentile), $138.60 to $984.50 (99th percentile), $984.60 to $7209.00 (>99th percentile), and $7209.00 to $19 798.00 (the 4 largest amounts).

Figure 1 gives the prevalence of promotion of OUD medications directed to physicians in 2014 and 2017. The diffusion of promotion across US counties was relatively slow during this period; 940 counties received some promotion in 2014, whereas 1193 counties received such promotion in 2017. The maps also indicate that most counties received small payment amounts per recipient; 2770 of 3130 (88.5%) counties in 2014 and 2865 of 3133 (91.4%) counties in 2017 received no payments or less than $78.60 per recipient. The distribution of this measure was highly skewed because of a small set of counties that received larger payment amounts (29 counties in 2014 and 34 counties in 2017 received payment amounts with a median value of $1785.10 [range, $984.50-$19 798.00] per recipient).

**Figure 2.**
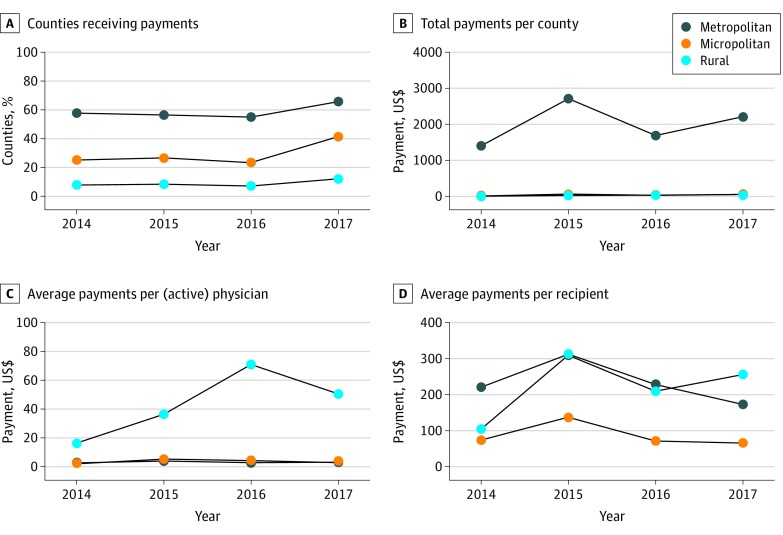
Promotion of Medications for Treating Opioid Use Disorder (OUD) by Rurality County-level data on pharmaceutical promotion of medications for treatment of OUD from the Sunshine Act’s Open Payments repository. A and B, Total sample was composed of 3140 counties: 1333 rural, 641 micropolitan, and 1166 metropolitan. B, Micropolitan line perfectly overlays the rural line visually. C and D, Payment amounts for 1458 counties where physicians received payments for OUD medications.

Figure 2 compares raw data of promotion for OUD medications over time for the 3 categories of rurality. The prevalence of promotion increased from 30.0% of counties in 2014 to 38.1% in 2017, primarily because of increases in the number of micropolitan counties receiving payments. The percentage of the 641 micropolitan counties that received payments increased from 25.3% (162 of 641 counties) in 2014 to 41.5% (266 counties) in 2017 (Figure 2A). A total of 1167 of 1329 rural counties (87.8%) did not receive any promotion from manufacturers in 2017, whereas 1219 rural counties (92.0%) did not have such promotion in 2014. In contrast, 765 of 1166 metropolitan counties (65.7%) received promotions in 2017.

We found higher payment amounts for OUD medications overall in urban counties than in rural counties in association with a larger portion of rural counties not receiving any payments as well as fewer physicians practicing in rural areas (Figure 2B). To separately account for these factors, we excluded counties not receiving any payments and presented the mean payment amounts per physician population in the remaining counties in Figure 2C and D. Table 1 presents descriptive statistics of the 3140 counties in this study and provides comparisons across rural, micropolitan, and metropolitan counties. After adjusting for the total number of active physicians (including primary care physicians) in a county (Figure 2C), the data show that the mean (SD) payments were highest ($43.70 [$121.60] per physician) in rural counties compared with micropolitan ($3.50 [$8.30] per physician) and metropolitan counties ($3.30 [$7.80] per physician). There was a statistically significant difference in the rural and urban payments weighted by active physicians (mean, $40.40; 95% CI, $4.00-$76.80) There were at least 72 times more physicians in urban counties (745.9 physicians; interquartile range, 20.0-556.0) compared with rural counties (10.3 physicians; interquartile range, 2.0-12.0) (Table 1). In Figure 2D, we divided the payment amounts by the number of physician recipients. There was no statistically significant difference in the payment amounts per recipient of OUD medication promotions in rural and metropolitan counties (mean difference, –$6.20; 95% CI, –$158.50 to $146.10). From 2014 through 2017, a rural-based physician recipient received $224.90 per year whereas an urban-based physician recipient received $231.10 per year (Table 1). These results suggest that among remaining counties that received any payments, a typical rural-based recipient did not receive any more or fewer payments than their urban-based counterparts.

**Table 1.** Pharmaceutical Promotion, Provider Supply, and Socioeconomic Characteristics for 3140 US Counties^a^

Characteristic	Counties, Mean or Median (IQR)			
	All	Rural	Micropolitan	Metropolitan
Observations, county-years	12 521	5303	2560	4658
Pharmaceutical promotion of OUD medications				
Likelihood of receiving promotion, %	31.6 (0-100)	8.98 (0)	29.3 (0-100)	58.7 (0-100)
Total payments of OUD medications, No.	0 (0-31.0)^b^	0 (0)^b^	0 (0-14.5)^b^	37.6 (0-346.5)^b^
Full sample				
Payments per physician, $	0 (0-0.3)^b^	0 (0)^b^	0 (0-0.2)^b^	0.17 (0-1.0)^b^
Payments per recipient, $	0 (0-19.1)^b^	0 (0)^b^	0 (0-13.7)^b^	19.4 (0-71.8)^b^
Payments per 100 000 residents, $	0 (0-48.5)^b^	0 (0)^b^	0 (0-31.4)^b^	34.8 (0-193.6)^b^
Subset of counties				
Payments per physician, $	0.89 (0.34-2.59)^b^	2.55 (1.05-6.82)^b^	0.81 (0.32-2.01)^b^	0.75 (0.39-2.20)^b^
Payments per recipient, $	49.58 (21.75-107.42)^b^	23.68 (15.17-75.41)^b^	29.48 (16.57-64.94)^b^	59.66 (29.14-125.00)^b^
Payments per 100 000 residents, $	145.28 (57.92-367.11)^b^	178.01 (80.75-482.34)^b^	99.10 (44.33-244.40)^b^	153.47 (60.39-397.74)^b^
Clinician^c^				
Active physicians, No.	294.0 (5.0-97.5)	10.3 (2.0-12.0)	59.5 (20.0-76.0)	745.9 (20.0-556.0)
Primary care physicians, No.	77.2 (4.0-40.0)	6.7 (2.0-9.0)	25.2 (12.0-33.0)	186.2 (12.0-173.0)
Primary care physicians per 100 000 residents, No.	52.8 (29.9-70.4)	44.2 (22.5-58.8)	55.5 (38.8-69.7)	61.1 (35.5-81.1)
Buprenorphine waivers per 100 000 residents, No.	5.95 (0-8.72)	4.00 (0-4.53)	6.43 (0-8.64)	7.91 (0.91-11.33)
Substance abuse treatment facilities per 100 000 residents, No.	4.43 (0-5.90)	5.32 (0-7.91)	4.61 (1.61-6.55)	3.33 (1.13-4.52)
Opioid-related deaths per 100 000 residents, No.	13.1 (3.49-18.4)	11.1 (0-16.5)	13.4 (5.72-18.5)	15.1 (7.79-19.9)
Race/ethnicity				
White population, %	77.0 (65.4-93.0)	79.4 (67.5-94.6)	77.2 (66.3-92.5)	74.3 (63.4-89.4)
Non-Hispanic African American population, %	8.94 (0.64-10.2)	7.56 (0.46-4.80)	8.16 (0.80-6.60)	10.9 (1.50-14.8)
Hispanic American population, %	9.08 (2.10-9.30)	8.02 (1.79-6.80)	10.2 (2.10-10.4)	9.68 (2.70-10.7)
Asian, Pacific Islander, or American Indian population, %	3.72 (1-3.20)	3.95 (0.80-2.40)	3.24 (1-2.90)	3.72 (1.27-4.30)
Socioeconomic characteristics				
Household income, $1000	47.8 (39.6-53.4)	43.2 (36.6-49.0)	45.5 (39.4-50.6)	54.4 (44.5-60.7)
Insured adults, 18-64 y of age, %	83.6 (78.9-89.0)	82.3 (77.4-88.0.)	83.5 (78.8-89.1)	85.0 (80.7-90.1)
Adults >64 y of age, %	17.8 (14.9-20.2)	19.9 (17.1-22.5)	17.1 (15.0-18.9)	15.8 (13.2-17.8)
County population, No. per 100 000 residents	1.02 (0.11-0.68)	0.1 (0.1-0.2)	0.43 (0.25-0.55)	2.35 (0.32-2.22)

Abbreviations: IQR, interquartile range; OUD, opioid use disorder.

^a^ We analyzed data from the Sunshine Act’s Open Payments, county-level opioid-related mortality rates from the National Vital Statistics System, opioid prescription rates from the Centers for Disease Control and Prevention, waiver data from Drug Enforcement Agency Active Controlled Substances Act Registrants, and other county-level characteristics from the Robert Wood Johnson Foundation County Health Rankings files.

^b^ Median values.

^c^ Active physicians include any type of physician. This number is used as a weight in the analysis. Primary care physicians were a physician subset. This number was used as a control variable in the analysis.

### Main Results

In our regression analysis, model 1 of Table 2 presents the ORs of receiving any promotion of OUD medications in a logistic regression. Compared with metropolitan counties, rural counties had reduced odds of receiving promotion of OUD medications (OR, 0.57; 95% CI, 0.44-0.74; *P* < .001). In contrast, the odds of micropolitan counties receiving promotions were not statistically different compared with those of the metropolitan county receiving promotions (OR, 1.04; 95% CI, 0.85-1.28; *P* = .69). In an alternative linear probability model (data from Washington DC and Delaware were included), rural counties were less likely to receive promotions (mean difference, 25.1 percentage points; 95% CI, –28.2 to –22.0 percentage points; *P* < .001).

**Table 2.** Demographic Characteristics Associated With Direct-to-Physician Pharmaceutical Payments for Medications Prescribed for Opioid Use Disorder^a^

Characteristic	Model 1: Likelihood of Receiving Medication Promotion, Odds Ratio (95% CI)	Incidence Rate Ratio (95% CI)		
		Model 2: Payments per Recipient	Model 3: Payments per 1000 Physicians	Model 4: Payments per 100 000 Residents
Rurality				
Metropolitan	1 [Reference]	1 [Reference]	1 [Reference]	1 [Reference]
Micropolitan	1.04 (0.85-1.28)	0.49 (0.36-0.66)^b^	0.55 (0.40-0.76)^b^	0.60 (0.45-0.80)^b^
Rural	0.57 (0.44-0.74)^b^	0.24 (0.17-0.34)^b^	0.60 (0.41-0.88)^c^	0.51 (0.36-0.72)^b^
Clinicians				
Buprenorphine waivers per residents, No.	1.07 (1.05-1.08)^b^	1.10 (1.07-1.13)^b^	1.12 (1.09-1.14)^b^	1.13 (1.10-1.15)^b^
Substance abuse treatment facilities per residents, No.	1.00 (0.98-1.02)	1.01 (0.98-1.04)	0.99 (0.96-1.02)	1.01 (0.98-1.04)
Primary care physicians per 100 000 residents, No.	1.01 (1.00-1.01)^b^	1.01 (1.01-1.02)^b^	0.99 (0.99-1.00)^c^	1.01 (1.01-1.02)^b^
Opioid-related deaths per residents, No.	1.00 (1.00-1.01)	1.01 (1.00-1.02)	1.00 (0.99-1.02)	1.01 (1.00-1.02)
Race/ethnicity				
Hispanic American population, %	0.99 (0.98-1.00)	0.98 (0.96-1.00)^d^	0.98 (0.96-1.00)^d^	0.99 (0.97-1.00)
Non-Hispanic African American population, %	1.00 (0.99-1.00)	0.97 (0.95-0.98)^b^	0.97 (0.95-0.98)^b^	0.97 (0.96-0.99)^b^
Asian, Pacific Islander, or American Indian population, %	1.01 (0.99-1.02)	1.01 (0.99-1.03)	1.00 (0.98-1.03)	1.01 (0.99-1.04)
Socioeconomic characteristics				
Household income, $1000	1.00 (0.99-1.01)	1.01 (0.99-1.02)	1.00 (0.98-1.01)	1.00 (0.99-1.02)
Insured adults, 18-64 y of age, %	0.99 (0.96-1.02)	0.93 (0.90-0.97)^b^	0.96 (0.93-1.00)^e^	0.97 (0.93-1.01)^e^
Adults >64 y of age, %	1.03 (1.00-1.06)^e^	1.02 (0.97-1.06)	1.05 (1.00-1.10)^d^	1.05 (1.00-1.09)^d^
County population, No. per 100 000 residents	9.72 (6.95-13.6)^b^	1.26 (1.14-1.40)^b^	1.12 (1.05-1.19)^b^	1.19 (1.10-1.29)^b^
Dependent variable, mean (SD)	0.32 (0.46)	64.06 (500.71)	2591.84 (79 379.09)	222.06 (2232.69)

^a^ Four models used data from 3140 counties. State and year fixed effects were included to control for unobserved temporal and geographic factors. In model 1, counties in Washington DC (n = 1) and Delaware (n = 3) were not excluded in the logistic regression because of lack of variations.

^b^ *P* < .001.

^c^ *P* < .01.

^d^ *P* < .05.

^e^ *P* < .10.

Model 2 presents the results of our county-level model of dollar amounts from pharmaceutical promotion of OUD medications in all 3140 counties. Rural counties received fewer dollars per recipient population by approximately 75.9% compared with metropolitan counties (adjusted IRR, 0.24; 95% CI, 0.17-0.34; *P* < .001).

We conducted several sensitivity analyses to examine whether the results were sensitive for the population denominator used for the payment amounts (Table 2). We found negative associations between rurality and payments when using any of 3 denominators: number of recipients (model 2), number of total active physicians in a county (model 3), and overall population of residents (model 4). In particular, in model 3, rural counties received fewer dollars per active physician compared with metropolitan counties (adjusted IRR, 0.60; 95% CI, 0.41-0.88; *P* < .01). In model 4, rural counties also received fewer dollars per 100 000 residents compared with metropolitan counties (IRR, 0.51; 95% CI, 0.36-0.72; *P* < .001).

## Discussion

Our study examined the association between promotion of OUD medications and rurality. The prevalence of any promotion was markedly lower in rural counties than in urban counties, even after controlling for several factors potentially associated with promotion at the county level: population, income, insurance rates, opioid-related mortality, number of buprenorphine waivers or buprenorphine-waivered clinicians, race/ethnicity, and other socioeconomic characteristics. The intensity of promotion (mean value) also was lower in rural areas, whether measured as per recipients, per total physicians, or per resident. These associations are concerning given that pharmaceutical promotion is a potential route to increase medication prescribing for OUD in rural areas and rural areas have fewer prescribers compared with urban areas.^9^ Rural areas have had higher rates of hospitalization for prescription opioid overdoses compared with urban areas.^21^ At a minimum, pharmaceutical promotion may help remedy information gaps associated with the limited training received by physicians about treatment of substance use disorder.^20^ A recent study^19^ found that promotion of OUD medication was associated with significantly higher levels of prescribing. Although that study^19^ did not explore causal relationships, it suggested that pharmaceutical promotion can be used a potential lever to expand medication prescribing for OUD.

Pharmaceutical promotion may be less likely to occur in rural areas than in micropolitan or urban areas for several reasons. First, travels and interactions in areas with smaller populations (in terms of residents and physicians) are less likely to be profitable for pharmaceutical manufacturers than travels and interactions in areas with larger and more densely distributed populations. Second, previous studies have found fewer SAMHSA-waivered buprenorphine prescribers in rural areas,^9^ and pharmaceutical promotions may target health care clinicians with SAMHSA waivers or health care clinicians with a history of prescribing substitute medications because these characteristics are indicators of interest in treating OUD, which is a highly stigmatized disorder.

Despite the potential role of pharmaceutical promotion to expand medication prescribing for OUD, policy makers, prescribers, and patients may feel uncomfortable with pharmaceutical promotion, especially given the association of opioid analgesic marketing with the ongoing opioid overdose crisis.^15^ Academic promotion, or training of prescribers by academic faculty or public health workers, may help address concerns related to bias and profit seeking. For example, academic faculty or public health workers without a vested interest in the sales of OUD medications could educate physicians about the risks, benefits, and alternatives of these medications. Academic promotion may be less biased than pharmaceutical manufacturer–initiated promotion in 2 respects. First, academic promotion may more accurately present the treatment risks, and second, it may more accurately and comprehensively describe alternative treatments. The latter is particularly important given the existence of 3 substitute OUD medications (methadone, buprenorphine, and naltrexone) and the need to individualize treatment rather than prescribe the same treatment to all clients to optimize positive treatment outcomes. Although academic promotion has been used successfully to promote treatments for other chronic health conditions, such as atrial fibrillation, to our knowledge, no studies exist regarding the effectiveness of academic promotion for treatments of OUD.^32^

Despite its potential benefits, a major drawback of academic promotion is its more limited capacity to reach health care clinicians. For example, 1 study^33^ in Pennsylvania found that there were more than 900 times as many pharmaceutical promoters as academic promoters. Therefore, academic promotion may serve as an important complement to pharmaceutical promotion but is unlikely to serve as a substitute. However, as our study showed, even pharmaceutical promotion may be less likely to occur in rural than urban areas. Technology-assisted promotion, such as with distance-learning technology, is 1 approach to expanding both academic and pharmaceutical promotion in rural areas,^34^ but more research is needed about the feasibility and effectiveness of technology-assisted promotion of OUD medications.

### Limitations

This study has several limitations. First, the regression models may omit some variables associated with promotion that are not easily measured and obtained at the county level, such as clinicians’ attitudes toward promotion. If these omitted variables are considerably associated with county population, our estimates could be biased in that those reasons may account for the rural disadvantage that we found. In this study, we included many county-level control variables to mitigate this possibility. Nonetheless, we do not assume that all factors have been included, especially because our aim was not to provide causal estimates. We assumed in our analysis that urban promotion did not benefit rural areas. Even though pharmaceutical companies were unlikely to have strong financial incentives to provide promotion in rural areas, it was possible that promotion in urban areas could indirectly be associated with rural areas through telehealth mechanisms (eg, an urban health care clinician prescribes medication to a rural resident remotely or during travels by urban physicians to rural county clinics on some days of the week). However, more research is needed to ascertain any spillover effects of promotion in urban areas to rural areas.

Second, the Sunshine Act data^18^ that we used on promotion of OUD medications in this study may not fully capture all relevant direct-to-physician promotion activities. Nurse practitioners and physician assistants may prescribe buprenorphine and naltrexone^35^ and receive payments from manufacturers. If these payments or other transfers of value are not passed through a physician, reporting of such payments are not mandated in the Open Payments database.^18^ Because the option of nurse practitioners and physician assistants to prescribe buprenorphine through SAMHSA waivers was not available until early 2017, it is unlikely this data omission affected the period of this study substantially. However, given evidence in Spetz et al^35^ showing that substantial increases in waivers have occurred through nurse practitioners and physician assistants since 2017, it will be important to consider this caveat in future studies.

### Conclusions

The study findings suggest that rural counties receive less promotion for prescribing of OUD medications, buprenorphine and naltrexone, and that these differences in promotion of OUD medications may be associated with differential commercial costs and benefits of promotion in rural settings.
